# A predictive scoring model to select suitable patients for surgery on primary tumor in metastatic esophageal cancer

**DOI:** 10.1002/cnr2.1898

**Published:** 2023-09-13

**Authors:** Laiming Wei, Jing Xu, Xueyou Hu, Yu Xie, Gang Lyu

**Affiliations:** ^1^ School of Advanced Manufacturing Engineering Hefei University Hefei China; ^2^ Department of Oncology the First Affiliated Hospital of Anhui Medical University Hefei China; ^3^ School of Big data and Artificial Intelligence Chizhou University Chizhou China; ^4^ Institute of Artificial Intelligence Hefei Comprehensive National Science Center Hefei China

**Keywords:** advanced esophagus cancer, nomogram, predictive scoring model, surgery on primary tumor, X‐tile

## Abstract

**Background:**

Surgery on primary tumor (SPT) has been a common treatment strategy for many types of cancer.

**Aims:**

This study aimed to investigate whether SPT could be considered a treatment option for metastatic esophageal cancer and to identify the patient population that would benefit the most from SPT.

**Methods:**

Data from 18 registration sites in the Surveillance, Epidemiology, and End Results Program database (SEER database) were analyzed to select patients with metastatic esophageal cancer. Multivariate Cox regression analysis was used to identify potential risk factors for pre‐treatment survival. Variables with a *p*‐value of less than 0.05 were used to construct a pre‐treatment nomogram. A pre‐surgery predictive model was then developed using the pre‐surgery factors to score patients, called the “pre‐surgery score”. The optimal cut‐off value for the “pre‐surgery score” was determined using X‐tile analysis, and patients were divided into high‐risk and low‐risk subsets. It was hypothesized that patients with a low “pre‐surgery score” risk would benefit the most from SPT.

**Results:**

A total of 3793 patients were included in the analysis. SPT was found to be an independent risk factor for the survival of metastatic esophageal cancer patients. Subgroup analyses showed that patients with liver or lung metastases derived more benefit from SPT compared to those with bone or brain metastases. A pre‐treatment predictive model was constructed to estimate the survival rates at one, two, and three years, which showed good accuracy (C‐index: 0.705 for the training set and 0.701 for the validation set). Patients with a “pre‐surgery score” below 4.9 were considered to have a low mortality risk and benefitted from SPT (SPT vs. non‐surgery: median overall survival (OS): 24 months vs. 4 months, HR = 0.386, 95% CI: 0.303–0.491, *p* < 0.001).

**Conclusion:**

This study demonstrated that SPT could improve the OS of patients with metastatic esophageal cancer. The pre‐treatment scoring model developed in this study might be useful in identifying suitable candidates for SPT. The strengths of this study include the large patient sample size and rigorous statistical analyses. However, limitations should be noted due to the retrospective study design, and prospective studies are needed to validate the findings in the future.

## 1 INTRODUCTION

Esophageal cancer ranked seventh among all cancers in terms of morbidity, causing a significant number of cancer‐related deaths globally.^1^ Symptoms of early esophageal cancer were often not obvious, leading to a majority of patients being diagnosed at middle or late stages when curative surgery is no longer an option.^1^ The five‐year survival rate for esophageal cancer was merely about 20%, particularly for those with distant spread.^1^

The main treatment strategy for metastatic esophageal cancer was chemotherapy, with limited progress in targeted drug therapies. Trastuzumab and Ramucirumab were the main targeted drugs for esophageal adenocarcinoma. Nimotuzumab has shown efficacy in esophageal squamous cell carcinoma. In terms of small molecule targeted drugs, only apatinib was available for esophageal adenocarcinoma in China.^2^, ^3^ The Chinese Society of Clinical Oncology guidelines recommended a few small molecule targeted drugs, such as apatinib and anlotinib, for esophageal squamous cell carcinoma.^3^, ^4^ In recent years, immunotherapy with Programmed Cell Death Protein 1 (PD‐1)/Programmed Cell Death‐Ligand 1 (PD‐L1) drugs have shown promising results in significantly prolonging overall survival (OS) in esophageal cancer patients.^5^ The overall condition of the patient was crucial in treatment management, and local therapies like surgery on the primary tumor (SPT) could relieve symptoms and improve the quality of life for metastatic cancer patients.^6^

Previous studies have presented conflicting views on the value of SPT in esophageal cancer. Some studies found no difference in prognosis between the SPT and non‐resection groups, while others reported promising outcomes with multimodal treatment including palliative resection, radiotherapy, and chemotherapy. Toshiaki Tanaka et al. conducted a study on the treatment methods and survival outcomes of 80 patients with metastatic esophageal squamous cell carcinoma. The study results showed that there was no significant difference in prognosis between the group of patients who underwent surgical primary tumor resection (SPT group) and the group who did not undergo resection (non‐resection group).^7^ According to the study conducted by Sahar A. Saddoughi et al., the survival of 52 patients with advanced esophageal cancer who underwent surgical primary tumor resection (SPT) in a local medical institution was recorded. The patients were followed up for a median duration of 324 days, ranging from 4 days to 8.5 years. The study found that the median overall survival (OS) of these patients was only 10.8 months. Based on these findings, the authors did not recommend surgical primary tumor resection as a treatment option for advanced esophageal cancer.^8^ However, it is worth noting that several articles have indicated that metastatic esophageal cancer patients can achieve favorable outcomes when treated with a multimodal approach, which typically includes palliative resection, radiotherapy, and chemotherapy. This comprehensive treatment strategy aims to alleviate symptoms, control the progression of the disease, and improve overall survival rates. It is important to consider individual patient characteristics, disease stage, and response to treatment when determining the most appropriate approach for managing metastatic esophageal cancer.^9^, ^10^

This study utilized the Surveillance, Epidemiology, and End Results Program database (SEER database), which collects real‐world cancer information for the U.S. population. The objective was to investigate the prognostic value of SPT in metastatic esophageal cancer using the SEER database and develop a scoring model to identify suitable candidates for surgery.

## 2 MATERIALS AND METHODS

### 2.1 Patients

This paper used SEER*Stat (version 8.3.5, Information Module: Diagnosed Patients and Their Treatment Patterns from 2004 to 2015) to identify patients with metastatic esophageal cancer. Metastatic esophageal cancer was defined as esophageal cancer which has spread to distant organs (e.g., lung, liver, bone, or brain). SPT was defined as direct surgery of cancer at the primary site, excluding destruction of local tumor (for instance, photodynamic therapy, electrocautery, laser, cryosurgery, excisional biopsy, polypectomy, and electrocautery). The inclusion criteria for patients were as follows: 1. the patient was diagnosed with metastatic esophageal cancer; 2. coexistence of primary tumor and metastatic tumor, with the metastases being unresectable; 3. esophageal cancer confirmed as the only primary tumor by histography; 4. clear information on initial treatment; 5. clear survival status. Baseline information and initial treatments were collected, including age at diagnosis, race, gender, tumor grade, primary tumor site, tumor size, histopathological type, regional lymph node involvement, distant metastasized organs, overall survival (OS), SPT, primary tumor radiotherapy, and chemotherapy. The following abbreviates were used for tumor grade: G1: Well differentiated (low grade), G2: Moderately differentiated (intermediate grade), G3: Poorly differentiated (high grade), G4: Undifferentiated (high grade).

Patients who met certain exclusion criteria were not included in the study: 1. the patients with unknown status of death or survival months; 2. the surgical status of the patient was unknown, or the patient accepted local tumor destruction (e.g., photodynamic therapy, electrocautery therapy), cryosurgery, laser, polypectomy, electrocautery, excisional biopsy), or combined metastases resection; 3. baseline information (e.g., race, location, grade and metastases) was unclear; 4. histopathology of the tumor, or the pathological type was neither squamous cell carcinoma nor adenocarcinoma; 5. patients were diagnosed with locally advanced cancer.

### 2.2 Statistical analysis

To construct a predictive model, multivariate analysis was used to identify independent risk factors. Multiple linear regression analysis was performed to evaluate the relationship between the multiple independent variables. Tolerance (TOL) method and variance inflation factor (VIF) method were adopted as the diagnosis methods of multicollinearity. TOL = 1/VIF, TOL value was between 0 and 1. When TOL was closer to 1, it indicted that the multicollinearity between the independent variables was weaker. Survival analysis was conducted using Kaplan–Meier (K‐M) curves, log‐rank tests, and Cox proportional hazards regression. The patients were randomly divided into training and validation groups. A predictive nomogram was constructed based on the retrospective coefficients of the selected independent prognostic factors.

We developed nomograms using retrospective coefficients of independent prognostic factors to assess survival. These nomograms allow for the calculation of a “survival score” based on the assigned points for each variable. In the nomogram, each variable corresponds to an integral on the top integral line intersected by a vertical line, indicating its contribution to survival. Points assigned to variables range from 0 to 10, and the sum of individual points determines the “survival score” or risk rating. To reduce overfitting bias, we internally validated the nomogram using bootstrap self‐sampling. We calculated the C‐index to evaluate the predictive ability of our nomogram in distinguishing predicted survival from actual survival. A C‐index value of 0.5 indicates that the nomogram fails to discriminate actual survival, while a value of 1.0 indicates perfect discrimination. Furthermore, we constructed calibration curves to assess the accuracy of our nomogram. These curves compare observed frequencies of true outcomes with predicted probabilities. A calibration curve that closely aligns with the 45‐degree line suggests a better fit of the nomogram's predictions.

In this study, we developed a pre‐treatment predictive model based on pre‐treatment prognostic factors. The resulting score was referred to as the “pre‐treatment survival score”. Additionally, we defined a “pre‐surgery score” which consisted of pre‐surgery factors in the pre‐treatment prediction model. Using X‐tile analysis, we divided patients in the training group into high‐risk and low‐risk subsets based on their “pre‐surgery scores”. We then compared the survival outcomes between surgical and non‐surgical patients in different risk subsets. Our assumption was that patients with low‐risk scores would have the most favorable prognosis, while there would be a significant difference in survival between the high‐risk and non‐surgical groups. Therefore, we utilized the “pre‐surgery score” calculated by the pre‐treatment predictive model to identify patients who would benefit from surgical treatment (SPT).

For χ2 analysis, cox regression analysis, multiple linear regression, and K‐M curve drawing, we utilized SPSS version 24.0 (SPSS, Chicago, IL). For nomogram construction, we used R studio software of R software version 4.1.2. All *p*‐values were considered two‐tailed, and statistical significance was determined at a *p*‐value below 0.05.

## 3 RESULTS

### 3.1 Patients' characteristics

**FIGURE 1:**
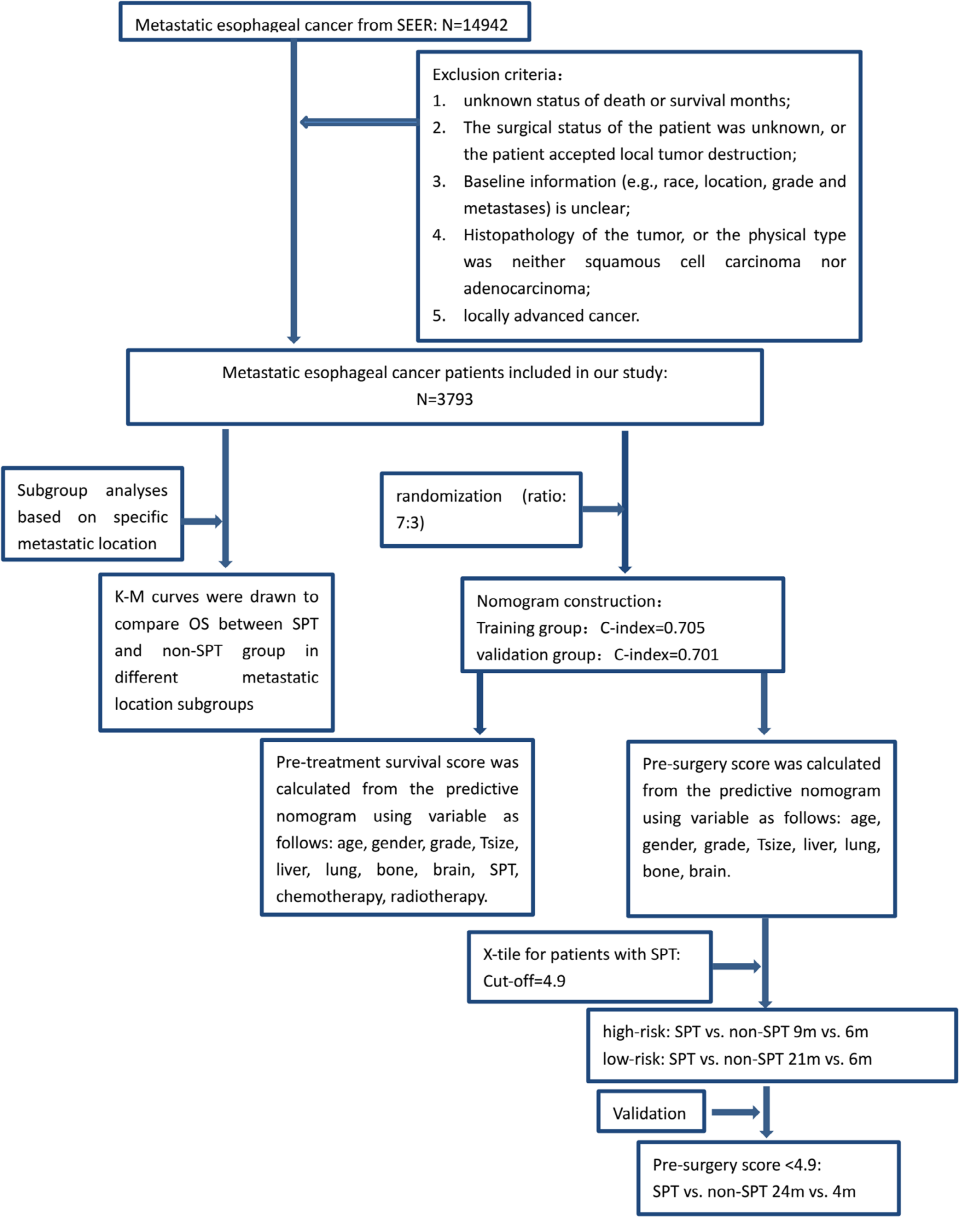
The flow chart of our study.

We retrieved 14 942 patients with metastatic esophageal cancer from the SEER database. After excluding patients with incomplete pre‐surgery information, a total of 3793 patients were included for analysis. (Figure 1). The median age of the patients was 63.92, and they were divided into two age subgroups: below 63 years and above 63 years. Multivariate analysis was performed to identify independent prognostic factors, including gender, primary tumor site, tumor grade, tumor size, distant metastases, and treatment choice (Table 1). It was found that younger age (≤63 years old), female patients, small tumors (≤5 cm), high and moderately differentiated tumors, and aggressive therapeutic intervention were significantly associated with better prognosis. In addition, according to collinearity analysis, all TOL values of the covariates were greater than 0.1, and VIF values were less than 10 (Table 2). Therefore, there was no significant multicollinearity among the covariates (age, gender, tumor grade, tumor size, different metastatic organs, SPT, radiotherapy, chemotherapy). In other words, the analysis confirmed that the model met the conditions for multiple linear regression.

**TABLE 1:** Multivariate cox analyses of potential preoperative risk factors for survival.

	Pre‐treatment (all patients)			Training set		
	No.	HR (95%CI)	*p value*	No.		*p value*
Characteristics	(*n* = 3793)			(*n* = 2661)	HR (95.0% CI)	
Age			0.396			0.036
≤63	1904	1		1331	1	
>63	1889	1.03 (0.962,1.103)		1330	1.091 (1.006,1.185)	
Race			0.863			0.892
White	3189	1		2235	1	
Black	384	1.032 (0.914,1.166)		265	1.033 (0.89,1.198)	
Other	220	0.993 (0.858,1.148)		161	1.025 (0.863,1.216)	
Gender			0.007			0.013
Male	3201	1		2238	1	
Female	592	0.878 (0.798,0.966)		423	0.866 (0.772,0.97)	
Site			0.487			0.925
Cervical/upper third of the thorax	205	1		139		
Middle/lower third of thorax/abdominal	3588	0.947 (0.811,1.105)		2522	1.009 (0.837,1.217)	
Histology			0.331			0.399
SCC	1063	1		747	1	
Adenocarcinoma	2730	0.957 (0.875,1.046)		1914	0.955 (0.858,1.063)	
Grade			<0.001			<0.001
G1/2	1586	1		1123	1	
G3/4	2207	1.22 (1.14,1.305)		1538	1.269 (1.17,1.377)	
Tumor size			0.001			0.005
≤5 cm	1934	1		1365	1	
>5 cm	1859	1.123 (1.05,1.2)		1296	1.121 (1.0351.214)	
LN			0.074			0.479
Negative	881	1		618	1	
Positive	2912	0.93 (0.86,1.007)		2043	0.966 (0.879,1.063)	
Liver			<0.001			<0.001
No	2935	1		2078	1	
Yes	858	1.236 (1.137,1.343)		583	1.231 (1.112,1.362)	
Lung			0.001			0.011
No	3273	1		2295	1	
Yes	520	1.188 (1.076,1.311)		366	1.166 (1.036,1.313)	
Bone			<0.001			<0.001
No	3370	1		2360	1	
Yes	423	1.305 (1.174,1.45)		301	1.274 (1.123,1.445)	
Brain			0.052			0.128
No	3690	1		2598	1	
Yes	103	1.222 (0.998,1.497)		63	1.223 (0.944,1.586)	
SPT			<0.001			<0.001
No	3408	1		2379	1	
Yes	385	0.468 (0.404,0.542)		282	0.457 (0.384,0.543)	
Radiotherapy			0.016			0.185
No	3335	1		2319	1	
Yes	458	0.85 (0.744,0.97)		342	0.9 (0.77,1.052)	
Chemotherapy			<0.001			<0.001
No/NA	1107	1		783	1	
Yes	2686	0.326 (0.302,0.352)		1878	0.318 (0.29,0.349)	

**TABLE 2:** The results of collinearity analysis.

Model	Non‐standardized coefficient		Standardized coefficient	*t*	Sig.	Multicollinearity	
	B	Standard error	Beta			TOL	VIF
(Independent variable)	48.405	4.446		10.886	0.000		
Age	−1.396	0.524	−.039	−2.665	0.008	0.965	1.036
Gender	1.201	0.715	.025	1.679	0.093	0.982	1.018
Grade	−2.283	0.523	−0.064	−4.363	0.000	0.994	1.006
Tumor size	−1.347	0.518	−0.038	−2.599	0.009	0.986	1.014
Liver	2.215	0.644	0.052	3.440	0.001	0.912	1.097
Lung	1.289	0.780	0.025	1.652	0.099	0.920	1.087
Bone	3.208	0.837	0.057	3.833	0.000	0.954	1.049
Brain	2.746	1.607	0.025	1.709	0.088	0.970	1.031
SPT	−14.073	1.105	−0.240	−12.738	0.000	0.594	1.682
Radiotherapy	−4.933	1.035	−0.091	−4.768	0.000	0.582	1.718
Chemotherapy	−8.938	0.584	−0.230	−15.304	0.000	0.939	1.065

*Note*: Dependent variable: Survival months.

Abbreviations: Sig, significance; TOL, tolerance; VIF, variance inflation factor.

**FIGURE 2:**
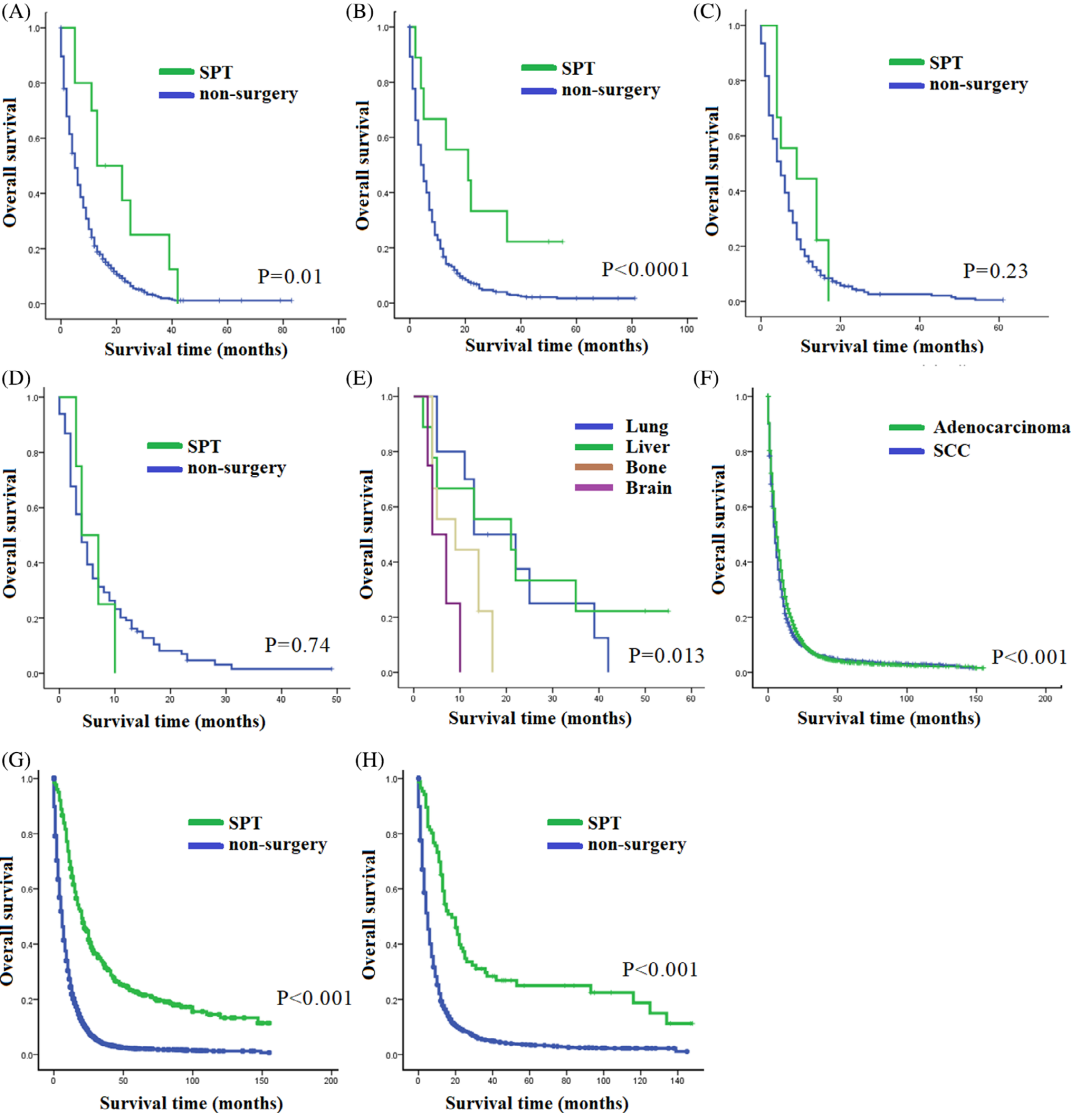
Kaplan–Meier (K‐M) curves of overall survival. The K‐M curves compared the overall survival between the SPT group and the non‐surgery group in various metastatic subgroups and different types of esophageal cancer: (A) The K‐M curves compared the overall survival between the SPT group and the non‐surgery group in patients with distant metastasis to the liver, (B) The K‐M curves compared the overall survival between the SPT group and the non‐surgery group in patients with distant metastasis to the lung, (C) The K‐M curve compared the overall survival between the SPT group and the non‐surgery group in patients with distant metastasis to the bone, (D) The K‐M curve compared the overall survival between the SPT group and the non‐surgery group in patients with distant metastasis to the brain, (E) The K‐M curves showed the overall survival in patients who underwent SPT based on different metastatic locations, (F) The K‐M curves compared the overall survival between patients with esophageal adenocarcinoma and esophageal squamous cell cancer (SCC), (G) The K‐M curve compared the overall survival between the SPT group and the non‐surgery group in the esophageal adenocarcinoma subgroup, (H) The K‐M curve compared the overall survival between the SPT group and the non‐surgery group in the SCC subgroup.

In various subgroups of metastatic cancer patients, the impact of SPT varied based on the specific metastatic locations. The results showed that liver metastases (HR = 0.45, 95%CI (0.23, 0.86), *p* = 0.01, Figure 2A) and lung metastases (HR = 0.37, 95%CI (0.18, 0.79), *p* < 0.0001, Figure 2B) were associated with a significant benefit from SPT. On the other hand, the benefit was not statistically significant for bone metastases (HR = 0.65, 95%CI (0.32, 1.32), *p* = 0.23, Figure 2C) or brain metastases (HR = 1.19, 95%CI (0.43, 3.26), *p* = 0.74, Figure 2D). Furthermore, the study demonstrated that patients with liver or lung metastases experienced a more substantial survival benefit from SPT. Median OS for patients with liver metastases was 13 months (2.511–23.489) with SPT, while for lung metastases it was 21 months (0–44.374) with SPT. In comparison, patients with bone metastases had a median OS of 9 months (0–20.687), and patients with brain metastases had a median OS of 4 months (0.080–7.920) (Figure 2E). In addition, although histology was not a significant prognostic factor for patients' survival (HR = 0.957, 95%CI (0.875,1.046), *p* = 0.331), our study showed that patients diagnosed with esophageal adenocarcinoma had a prolonged OS compared to those with esophageal squamous cell carcinoma (SCC) (median OS of adenocarcinoma vs. SCC: 6 months vs. 5 months, *p* < 0.001, Figure 2F). Furthermore, patients who underwent SPT had longer OS compared to those who did not undergo surgery in both the adenocarcinoma subgroup and SCC subgroup (median OS for adenocarcinoma: SPT vs. non‐SPT: 20 months vs. 6 months, *p* < 0.001, Figure 2G; median OS for SCC: SPT vs. non‐SPT: 18 months vs. 5 months, *p* < 0.001, Figure 2H).

### 3.2 Construction of the predictive model

**FIGURE 3:**
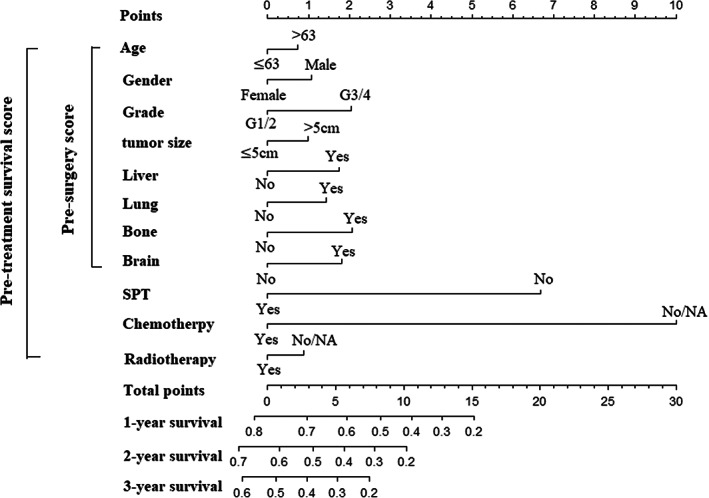
Pre‐treatment nomogram for patients based on pre‐treatment risk factors.

After selecting a training group consisting of 2661 patients and a validation group with the remaining patients (randomization ratio: 7:3), the constructed predictive model incorporated the identified risk factors. The pre‐treatment nomogram, which was a graphical representation of the predictive model, was illustrated in Figure 3 based on the training group. The nomogram provided a tool for estimating an individual patient's prognosis based on the identified risk factors and their associated weights in the predictive model.

**FIGURE 4:**
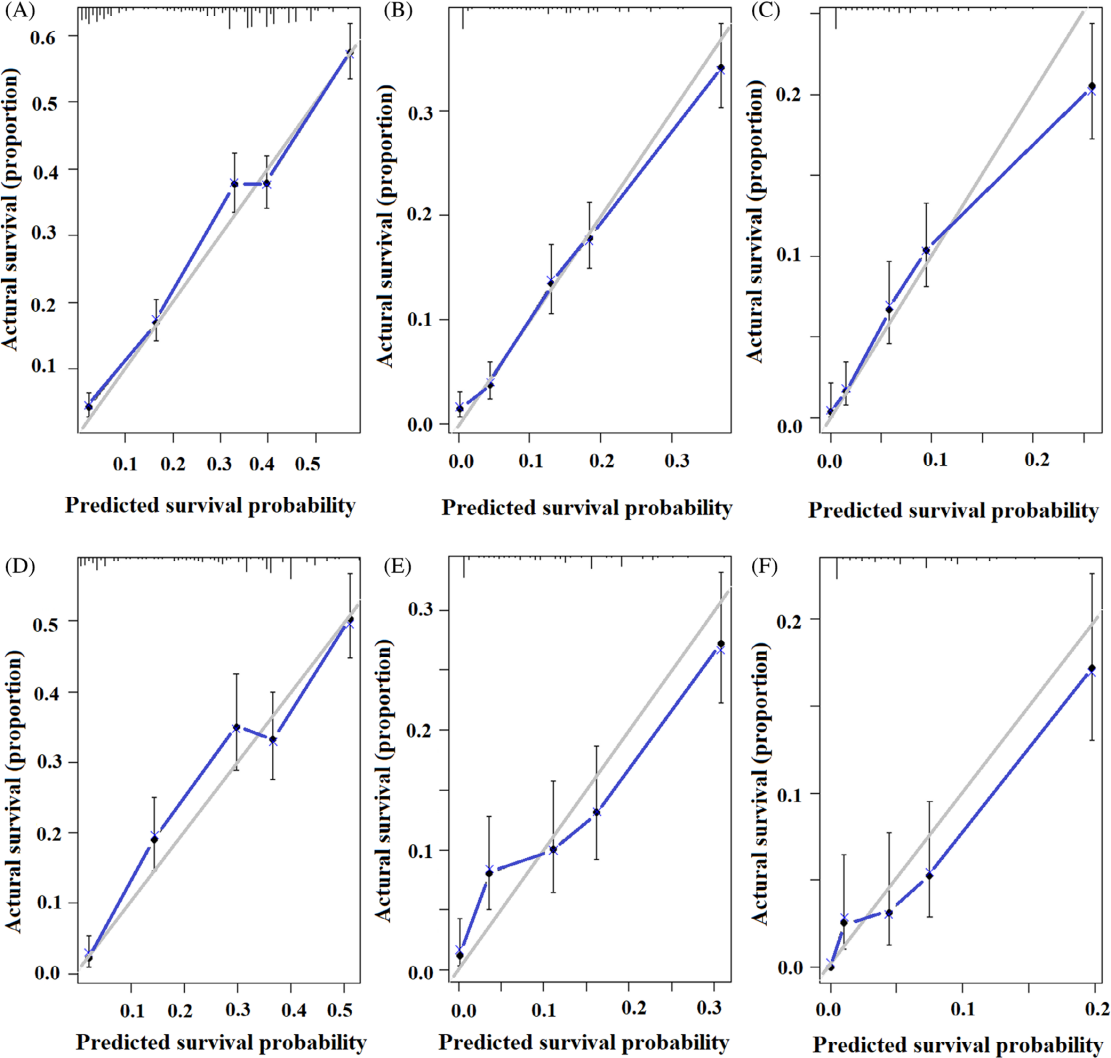
Calibration curves for survival prediction of pre‐treatment nomogram: 1‐year (A), 2‐years (B) and 3‐years (C) of overall survival (OS) for the training group, calibration curves for the OS prediction at 1 year (D), 2‐years (E) and 3‐years (F) in the validation group.

The performance of the predictive model was evaluated using the C‐index, which measures the concordance between the predicted and observed survival. In the training set, the C‐index was found to be 0.705, indicating a moderate level of accuracy in predicting patient survival. Similarly, in the validation set, the C‐index was 0.701, suggesting consistent performance and generalizability of the model. To further assess the calibration or accuracy of the nomogram's predictions, calibration curves were constructed. These curves compare the predicted survival probabilities from the nomogram with the actual observed patient survival. The calibration curves demonstrated good agreement between the predicted and observed survival in Figure 4. The “pre‐treatment survival score” was defined as the patient score calculated by the predictive model. This score serves as an indicator of the predicted OS of patients with metastatic esophageal cancer at specific time points, such as 1, 2, and 3 years. The pre‐treatment survival score can provide valuable information regarding the expected survival outcome for individual patients based on their specific characteristics and risk factors.

### 3.3 Development of pre‐surgery scoring rule to select patients suitable for SPT

**FIGURE 5:**
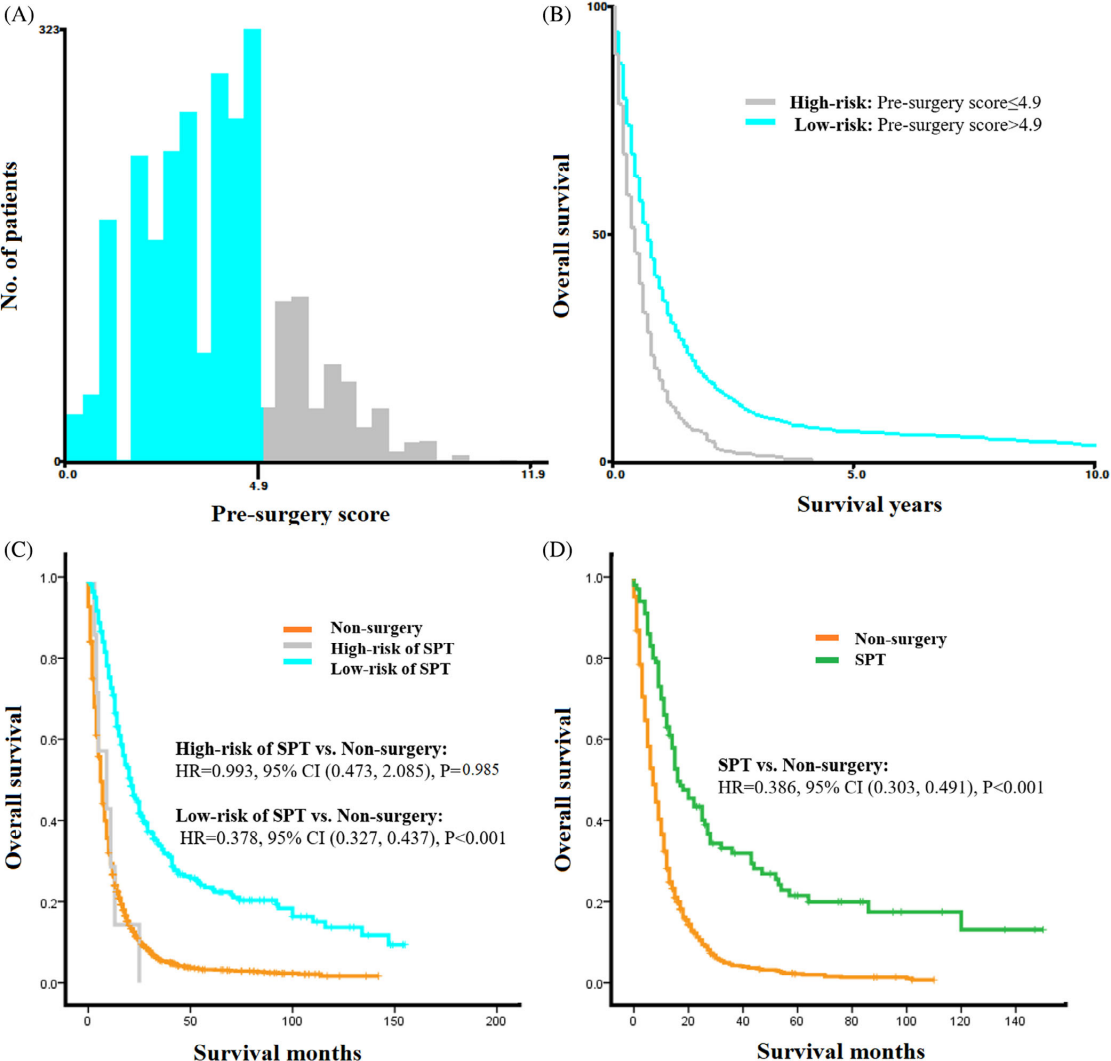
X‐tile analysis based on the pre‐surgery score and Kaplan–Meier (K‐M) curves for overall survival (OS). (A) Training set: Numbers of patients in high and low mortality risk subsets, (B) K‐M curves for OS in high and low risk subsets, (C) K‐M curves for OS in high and low risk subsets compared with that in non‐surgery group. (D) Low‐risk group from validation set: K‐M curves for OS of SPT and non‐surgery patients.

To select patients suitable for SPT, a pre‐surgery scoring rule was developed using non‐treatment factors from the predictive model. The patient's risk score, known as the “pre‐surgery score”, was calculated using non‐treatment factors (Figure 3). Patients in the training group were divided into high‐risk and low‐risk subsets based on their pre‐surgery scores, with a cut‐off value of 4.9 by X‐tile analysis (Figure 5A,B). The K‐M survival curves were utilized to evaluate the overall survival of these subsets. The results showed that patients in the low‐risk subset for SPT mortality had significantly longer overall survival compared to those who did not undergo surgery (21 months vs. 6 months, *p* < 0.001, Figure 5C). Conversely, high‐risk patients did not exhibit a significant difference in survival compared to the non‐surgery group (9 months vs. 6 months, HR = 0.993, 95%CI: 0.473–2.085, *p* = 0.985, Figure 5C). These findings suggested that patients in the low‐risk subset, as determined by their pre‐surgery scores, might benefit from SPT, while surgery might not confer a significant survival advantage for high‐risk patients.

The pre‐surgery scoring rule was further validated using the validation set, and it was found that patients in the low‐risk group benefited from SPT compared to those without surgery (SPT vs. non‐surgery: median OS: 24 months vs. 4 months, HR = 0.386, 95%CI: 0.303–0.491, *p* < 0.001, Figure 5D). Based on these analyses and findings discussed, if a patient's pre‐surgery score was lower than 4.9 points at the time of diagnosis, it was recommended to consider SPT as the primary treatment.

## 4 DISCUSSION

Up until now, SPT has not been recommended in the NCCN guidelines for metastatic esophageal cancer patients. The primary treatment options for these patients included systemic therapy, palliative supportive care, and participation in clinical trials. Systemic drug therapy could be prescribed for patients with newly diagnosed advanced metastatic esophageal cancer if tolerated. For metastatic esophageal cancer patients who experienced disease progression after systemic therapy, alternative regimens could be considered. In cases of local recurrence or distant metastases after radical treatment, systemic medical therapy was recommended if tolerated. It was important to note that treatment strategies might differ for metastatic esophageal squamous cell carcinoma and esophageal adenocarcinoma. Currently, the standard first‐line treatment for metastatic esophageal cancer involved the use of immune checkpoint inhibitors combined with chemotherapy.^6^ For patients with metastatic esophageal cancer, including both squamous cell carcinoma and adenocarcinoma, the recommended first‐line therapy was the cisplatin + fluorouracil chemotherapy regimen in combination with pembrolizumab.^6^ However, for patients with metastatic esophageal adenocarcinoma, the first‐line therapy could involve the addition of nivolumab to the oxaliplatin + fluorouracil regimen.^6^ On the other hand, for patients with metastatic esophageal squamous cell carcinoma, the recommended first‐line therapy was the combination of paclitaxel + cisplatin chemotherapy with carrelizumab. In cases where patients were not eligible for immune checkpoint inhibitors, chemotherapy alone might be considered. Common chemotherapy regimens for metastatic esophageal squamous cell carcinoma typically involved the use of cisplatin in combination with fluorouracil or paclitaxel plus platinum. Similarly, for metastatic esophageal adenocarcinoma, common chemotherapy regimens included cisplatin or oxaliplatin in combination with fluorouracils. In cases where patients had good physical fitness, a three‐drug combination regimen consisting of paclitaxel, platinum, and fluorouracil might also be considered as first‐line therapy. Additionally, for patients with HER‐2‐positive metastatic esophageal adenocarcinoma, the recommended first‐line therapy involved the addition of trastuzumab to the cisplatin + fluorouracil regimen.

Additionally, several studies have recommended primary resection for metastatic esophageal cancer. For example, Yin‐Kai Chao et al. investigated 54 esophageal cancer patients with distant lymph node metastasis.^9^ They discovered that patients who underwent R0 surgery on the primary tumor after neoadjuvant chemoradiotherapy had a median overall survival (OS) of 45 months.^9^ Even in the non‐surgical group where R0 resection was not achieved, the median survival time was 45 months.^9^ Patients who underwent surgery solely on the primary tumor exhibited a 3‐year disease‐free survival rate of 9%. Their median disease‐free survival time of 10.5 months was higher than that in the non‐surgical subset (9.5 months).^9^ Another study by Elke Van Daele et al. reviewed postoperative complications and OS in 12 metastatic esophageal cancer patients who underwent multidisciplinary treatment including surgery, with a median postoperative hospital stay of 15 days.^10^ Only one patient experienced radiographic anastomotic leakage, and there were no postoperative mortalities.^10^ After a median follow‐up time of 22 months, half of the surgically resected patients survived, and 33% had no recurrence of the disease.^10^ Kaplan–Meier curves indicated a promising likelihood of long‐term survival rate after aggressive multimodal treatment that included palliative surgery.^10^ Furthermore, Jiahua Lyu et al. investigated 141 patients with advanced esophageal cancer and found that the objective response rate of the primary tumor was significantly higher in patients who received chemoradiotherapy compared to those who received chemotherapy alone (74.5% vs. 45.3%, *p* = 0.001).^11^ In the subset receiving chemoradiotherapy, the 3‐year survival rate was 25.5%, which was higher than the 4.7% in the chemotherapy‐only subset.^11^ The median OS in the chemoradiotherapy subset was 14 months, which was also longer than the 11 months observed in the chemotherapy‐only subset.^11^ These findings aligned with our results mentioned earlier.

At the same time, case reports have also provided support for long‐term survival outcomes in advanced esophageal cancer patients following multidisciplinary treatment, including local therapy targeting the primary lesion. For example, Mudan S et al. reported a case of a patient with esophageal cancer and liver metastases who underwent simultaneous combined resection.^15^ They followed this case for 29 months and observed no tumor progression.^11^ Hiroyuki Isshiki reported a case of a 71‐year‐old patient with esophageal cancer and multiple liver metastases.^16^ After six courses of chemotherapy, the liver metastases disappeared, but the esophageal cancer component remained.^16^ Radiotherapy was used to treat the residual esophageal primary tumor.^12^ Our findings were consistent with these reports, showing significantly longer OS in patients who underwent SPT. The patient samples in our study were derived from the SEER database, which reflects real‐world outcomes for cancer patients.

Indeed, there was a growing body of evidence suggesting that patients with metastatic cancers can benefit from SPT, not limited to esophageal cancer but also in other types of cancer such as lung, colorectal, ovarian, breast, and kidney cancers.^13^, ^14^ The underlying mechanism by which SPT benefits metastatic cancer patients was based on the hypothesis that primary tumors often induce immunosuppression, which hampers the immune system's ability to respond to the tumor.^17^ Danna et al. reported that the presence of a primary tumor suppresses both T cell activation and antibody responses. However, after surgical resection of the primary tumor, the immune system was restored, even in the presence of metastatic tumors.^15^ Additionally, SPT can reduce the overall tumor burden in the patient's body, leading to more significant treatment outcomes with systemic therapy.^16^ It has been suggested that primary tumors have the potential to continuously disseminate tumor cells, which can contribute to further tumor metastasis. Surgical removal of the primary tumor eliminates this potential source of tumor dissemination.^17^

In addition, SPT provided local treatment for primary tumors, which can have clinical benefits in patients with advanced esophageal cancer. SPT could help alleviate symptoms such as esophageal obstruction, improve nutritional status, and relieve symptoms caused by regional lymph node metastasis and compression. Previous studies have demonstrated the value of SPT in patients with advanced incurable colorectal cancer^18^, ^19^ and gastric cancer,^20^, ^21^ where maintaining the patency of the digestive system was crucial for the quality of life of patients. Primary resection in this population can prevent tumor‐related complications such as bleeding, perforation, and obstruction. For patients with esophageal cancer, dysphagia was a major symptom that significantly impacts their quality of life. Tumors in the esophagus could also lead to perforation and bleeding. Palliative or radiological resection of the primary tumor can reduce the incidence of oncology‐related emergencies and improve overall patient well‐being. In a previous study, we found that patients who underwent SPT had a median overall survival (OS) of 20 months, which was nearly 10 months longer than patients who received radiotherapy alone.^12^ Ando et al. also reported that advancements in perioperative management and surgical techniques can improve survival outcomes in patients with advanced esophageal cancer.^22^

In our study, certain clinicopathological features before initial therapy, which refers to the first‐line treatment or the first treatment recommended for cancers, were found to be significantly associated with a better prognosis in patients. These features include younger age, female gender, smaller tumor size (≤5 cm), higher and moderately differentiated tumors, and aggressive therapeutic intervention. It was interesting that esophageal cancer appeared to be more common and have a worse prognosis in men compared to women. This observation might be attributed to various factors, including social pressure, male lifestyle habits such as smoking or drinking, and hormonal differences.^23^ In clinical practice, tumor‐lymph node‐metastasis (TNM) staging is commonly used to predict the survival rate of cancer patients. Our study suggested that our predictive model might serve as an important complement to TNM staging, particularly in selecting the optimal anti‐cancer treatment for patients with stage IV esophageal cancer at the time of diagnosis. Our previous study indicated that surgical treatment (SPT) or radiotherapy might be beneficial for the survival of patients with metastatic esophageal cancer, regardless of whether cancer‐specific survival (CSS) or overall survival (OS) was used as the study endpoint.^24^ Using OS as the endpoint in the study is a common and appropriate choice. OS is widely accepted as the primary efficacy endpoint in oncology clinical trials because it represents the ultimate outcome of interest ‐ the length of time patients survive from the start of treatment until death from any cause. In addition, using OS as the endpoint allowed for a more holistic assessment of treatment efficacy, considering the potential benefits and risks of various treatment modalities.^25^ It was commendable that our nomogram also demonstrated good performance in predicting outcomes for patients with metastatic esophageal cancer, in comparison to a study by Tang et al. that used CSS as the endpoint.^26^

Our study had several advantages, primarily due to the large number of patients included and the rigorous statistical analyses conducted. However, there were some limitations that should be acknowledged. Firstly, the patients included in this study were not randomized, which introduced the possibility of selection bias and the influence of unmeasured confounding variables. Additionally, the SEER database did not provide information on the specific type of surgery, such as emergency surgery or elective surgery, which could be important factors affecting patient outcomes. Postoperative complications are also critical risk factors for cancer patients, and their impact was not considered in this study. Future research should compare the postoperative mortality rates between these two types of surgery in metastatic esophageal cancer patients. Moreover, the SEER database lacked data on patients' general condition and other diseases. Performance status (PS) and symptoms, which are important considerations for surgical recommendations according to guidelines, were not included in this study. This led to increased uncertainty in the accuracy of our nomogram. Future studies should incorporate PS as one of the predictive factors to improve the predictive accuracy of the nomogram. Additionally, although we considered chemotherapy in the nomogram prediction model, different regimen choices might result in different prognostic outcomes. With the emergence of new treatments, such as immunotherapy, predicting survival for cancer patients might become more challenging due to the combination of different treatment options. This should be taken into account in future studies.

## 5 CONCLUSION

In conclusion, SPT has been shown to improve survival in patients with metastatic esophageal cancer, but patient selection is crucial. Our predictive model and pre‐surgery scoring system offer a valuable tool for identifying appropriate candidates for surgical treatment. If the patient's pre‐surgery score was lower than 4.9, SPT could be considered as one of the treatment methods. The advantages of our study rooted in the large number of patient and adequate statistical analyses. However, further researches involving prospective studies are needed to validate the effectiveness of our model.

## AUTHOR CONTRIBUTIONS

**Laiming Wei:** Conceptualization (equal); data curation (equal); formal analysis (equal); funding acquisition (equal); investigation (equal); methodology (equal); project administration (equal); resources (equal); software (equal); visualization (equal); writing – original draft (equal); writing – review and editing (equal). **Jing Xu:** Conceptualization (equal); data curation (equal); formal analysis (equal); investigation (equal); methodology (equal); resources (equal); software (equal); supervision (equal); validation (equal); visualization (equal); writing – original draft (equal); writing – review and editing (equal). **Xueyou Hu:** Funding acquisition (equal); writing – original draft (supporting); writing – review and editing (supporting). **Yu Xie:** Conceptualization (equal); supervision (equal); writing – review and editing (supporting). **Gang Lyu:** Conceptualization (equal); supervision (equal); writing – review and editing (supporting).

## CONFLICT OF INTEREST STATEMENT

The authors have stated explicitly that there are no conflicts of interest in connection with this article.

## ETHICS STATEMENT

NA. Only the publicly available databases have been used in this study.

## Data Availability

Data sharing is not applicable to this article as no new data were created or analyzed in this study.
